# Editorial: Molecular Ecology and Genetic Diversity of the *Roseobacter* Clade

**DOI:** 10.3389/fmicb.2018.01185

**Published:** 2018-06-01

**Authors:** Rolf Daniel, Meinhard Simon, Bernd Wemheuer

**Affiliations:** ^1^Genomic and Applied Microbiology and Göttingen Genomics Laboratory, Institute of Microbiology and Genetics, University of Göttingen, Göttingen, Germany; ^2^Biology of Geological Processes, Institute for Chemistry and Biology of the Marine Environment, University of Oldenburg, Oldenburg, Germany; ^3^Centre for Marine Bio-Innovation, The University of New South Wales, Sydney, NSW, Australia; ^4^School of Biological Earth and Environmental Sciences, The University of New South Wales, Sydney, NSW, Australia

**Keywords:** *Roseobacter* group, microbial ecology, microbial evolution, molecular ecology, microbial diversity

The *Roseobacter* clade, more recently referred to as *Roseobacter* group, is a paraphyletic group within the *Rhodobacteraceae* (*Alphaproteobacteria*) (Simon et al., 2017). It is one of the most widely distributed and abundant bacterial groups in the marine ecosystem constituting up to 30% of bacterial communities in pelagic environments. *Roseobacter* group members inhabit a great variety of marine habitats and niches. They exhibit a free-living or surface-associated lifestyle and even occur in oxic and anoxic sediments (Luo and Moran, 2014). They are physiologically and genetically very versatile. Some of the important functional traits found in the *Roseobacter* group are the utilization of various organic and inorganic compounds including the catabolism of dimethylsulfoniopropionate (DMSP), energy acquisition by sulfur oxidation, aerobic anoxygenic photosynthesis and carbon monoxide oxidation, and the production of secondary metabolites (Buchan et al., 2005; Wagner-Döbler and Biebl, 2006; Brinkhoff et al., 2008; Todd et al., 2012).

Although various aspects of the *Roseobacter* group have been studied in recent years (e.g., Luo and Moran, 2014; Wemheuer et al., 2014, 2017; Gram et al., 2015; Voget et al., 2015; Lutz et al., 2016; Zhang et al., 2016), our knowledge about its ecological significance and the evolutionary processes shaping the genomes of this group is still limited. The 10 publications presented in this research topic “Molecular Ecology and Genetic Diversity of the *Roseobacter* Clade” highlight new and interesting findings on the evolution, biodiversity, and functions of the *Roseobacter* group in the marine environment. Contributions include original research, a perspective, and a comprehensive review.

In three contributions, culture-independent approaches are employed to assess the abundance and distribution of *Roseobacter* group members in marine pelagic systems (Bakenhus et al.; Freese et al.) and Pacific sediments (Pohlner et al.). Bakenhus et al. highlight the major role of several pelagic members of the *Roseobacter* group in processing phytoplankton-derived organic matter, although this group constituted only a minor proportion of the total bacterioplankton community. Freese et al. show that a previously unknown, distinct group of *Phaeobacter gallaeciensis* possess a limited number of group-specific genes, which may be relevant for its association with mesozooplankton and for its colonization in marine pelagic systems.

As most studies on the abundance and diversity of the *Roseobacter* group were conducted on pelagic samples (e.g., Giebel et al., 2011; Wemheuer et al., 2015; Billerbeck et al., 2016), the distribution and function of this group in sediments is less understood (but see Kanukollu et al., 2015). In their contribution, Pohlner et al. demonstrate that different oligo- and ultraoligotrophic oceanic provinces in the subtropics and tropics of the Pacific were characterized by specific sediment communities and *Roseobacter* group members, distinct from those of the more productive temperate and subarctic regions. *Roseobacter*-affiliated OTUs were dominated by uncultured members, demonstrating the need to obtain cultured *Roseobacter* representatives from sediments to link community structures to specific metabolic processes at the seafloor.

Aside from community patterns, the functional response of the ambient bacterial community toward a *Phaeocystis globosa* bloom in the southern North Sea was studied using metaproteomic approaches (Wöhlbrand et al.) This study highlights the application of different sample preparation techniques and mass spectrometric methods for a comprehensive characterization of marine bacterioplankton responses to changing environmental conditions. The comprehensive approach verified previous metaproteomic studies of marine bacterioplankton (e.g., Sowell et al., 2011; Teeling et al., 2012; Georges et al., 2014), but also revealed new insights into carbon and nitrogen metabolism.

Gardiner et al. demonstrate for the first time temperature-dependent regulation of the RTX-like proteins in the important seaweed pathogen *Nautella italica* R11 and thus provides the basis for future functional studies on the temperature-dependent manner of secreted proteins and their role in pathogenicity and/or environmental persistence of *N. italica* R11. This is of crucial importance as increasing ocean temperatures associated with climate change are predicted to cause greater host stress and more extensive disease events in macroalgae.

Two studies focused on adaptations to environmental properties of the *Roseobacter* group (Bullock et al.; Ebert et al.) Ebert et al. describe for the first time a regulatory network solely composed of four Crp/Fnr-family regulators responsible for the metabolic adaptation to low oxygen tension observed in the marine bacterium *Dinoroseobacter shibae*. Bullock et al. review the evolution of DMSP metabolism in marine phytoplankton and bacteria, thereby illustrating that the enzymes of DMSP demethylation and cleavage pathways are examples of the various processes of enzyme adaptation and evolution, which occurred within the *Roseobacter* group in the last 250 million years.

*N*-acyl-homoserine lactones (AHLs) constitute the major class of semiochemicals in quorum sensing (QS) systems (Williams, 2007; Papenfort and Bassler, 2016). Complex mixtures of AHLs have been found for the several members of the *Roseobacter* clade (Wagner-Döbler et al., 2005). In their contribution, Doberva et al. discover an unsuspected capacity of the marine *Rhodobacteraceae* strain MOLA 401 to synthesize 20 different putative AHLs by a combination of biosensor-based screening and liquid chromatography coupled to mass spectrometry and nuclear magnetic resonance. The authors conclude that the higher diversity of signaling molecules, unusual for a single strain, shows new molecular adaptations of QS systems to planktonic life.

Horizontal gene transfer (HGT) is an important driver of bacterial diversification and the evolution of prokaryotic genomes (Polz et al., 2013; Sun et al., 2015). Two articles in this research topic highlight the importance of HGT in the *Roseobacter* group. Bartling et al. identified a *Roseobacter*-specific RepABC-type operon in the draft genome of the marine rhizobium *Martelella mediterranea* DSM 17316^T^, whereas Petersen and Wagner -Döbler provide the first evidence for conjugational plasmid transfer across biogeographical and phylogenetic barriers in the *Rhodobacteraceae*.

In summary, the articles presented in this research topic demonstrate the benefits of using multidisciplinary approaches to analyze and deepen our knowledge of the ecological significance, functions, and the evolutionary processes shaping the genomic basis and responses of the *Roseobacter* group to environmental conditions. Moreover, many challenges and questions were identified that remain to be addressed. We thank all the participating authors for their contributions, which we believe will be the basis for future investigations into the function, evolution, and diversity of the fascinating *Roseobacter* group.

## Author contributions

All authors listed have made a substantial, direct, and intellectual contribution to the work, and approved it for publication.

### Conflict of interest statement

The authors declare that the research was conducted in the absence of any commercial or financial relationships that could be construed as a potential conflict of interest.
